# Activation of GIPR Exerts Analgesic and Anxiolytic-Like Effects in the Anterior Cingulate Cortex of Mice

**DOI:** 10.3389/fendo.2022.887238

**Published:** 2022-05-30

**Authors:** Xin-shang Wang, Yong-li Jiang, Liang Lu, Ban Feng, Xue Ma, Kun Zhang, Shao-yu Guan, Le Yang, Qing-yu Fan, Xiao-chen Zhu, Fan Yang, Jing-yu Qi, Liu-kun Yang, Xu-bo Li, Ming-gao Zhao, Wen Jiang, Zhen Tian, Shui-bing Liu

**Affiliations:** ^1^ Department of Pharmacology, School of Pharmacy, Fourth Military Medical University, Xi’an, China; ^2^ Precision Pharmacy and Drug Development Center, Department of Pharmacy, Tangdu Hospital, Fourth Military Medical University, Xi’an, China; ^3^ Department of Neurology, Xijing Hospital, Fourth Military Medical University, Xi’an, China; ^4^ Department of Pharmacology, College of Pharmaceutical Sciences, Southwest University, Chongqing, China

**Keywords:** glucose-dependent insulinotropic polypeptide receptor, chronic pain, anxiety, neurotransmission, neuroinflammation, anterior cingulate cortex

## Abstract

**Background:**

Chronic pain is defined as pain that persists typically for a period of over six months. Chronic pain is often accompanied by an anxiety disorder, and these two tend to exacerbate each other. This can make the treatment of these conditions more difficult. Glucose-dependent insulinotropic polypeptide (GIP) is a member of the incretin hormone family and plays a critical role in glucose metabolism. Previous research has demonstrated the multiple roles of GIP in both physiological and pathological processes. In the central nervous system (CNS), studies of GIP are mainly focused on neurodegenerative diseases; hence, little is known about the functions of GIP in chronic pain and pain-related anxiety disorders.

**Methods:**

The chronic inflammatory pain model was established by hind paw injection with complete Freund’s adjuvant (CFA) in C57BL/6 mice. GIP receptor (GIPR) agonist (D-Ala^2^-GIP) and antagonist (Pro^3^-GIP) were given by intraperitoneal injection or anterior cingulate cortex (ACC) local microinjection. Von Frey filaments and radiant heat were employed to assess the mechanical and thermal hypersensitivity. Anxiety-like behaviors were detected by open field and elevated plus maze tests. The underlying mechanisms in the peripheral nervous system and CNS were explored by GIPR shRNA knockdown in the ACC, enzyme-linked immunosorbent assay, western blot analysis, whole-cell patch-clamp recording, immunofluorescence staining and quantitative real-time PCR.

**Results:**

In the present study, we found that hind paw injection with CFA induced pain sensitization and anxiety-like behaviors in mice. The expression of GIPR in the ACC was significantly higher in CFA-injected mice. D-Ala^2^-GIP administration by intraperitoneal or ACC local microinjection produced analgesic and anxiolytic effects; these were blocked by Pro^3^-GIP and GIPR shRNA knockdown in the ACC. Activation of GIPR inhibited neuroinflammation and activation of microglia, reversed the upregulation of NMDA and AMPA receptors, and suppressed the enhancement of excitatory neurotransmission in the ACC of model mice.

**Conclusions:**

GIPR activation was found to produce analgesic and anxiolytic effects, which were partially due to attenuation of neuroinflammation and inhibition of excitatory transmission in the ACC. GIPR may be a suitable target for treatment of chronic inflammatory pain and pain-related anxiety.

## Introduction

Pain is a multidimensional experience that includes sensory-discriminative, emotional-affective, and cognitive components (1). Acute pain is essential for safety and teaches human and animals to avoid potential or actual tissue damage (2). Chronic pain, or persistent pain, usually lasts for a prolonged period and can seriously affect the quality of life of those affected. Chronic pain has become a major medical problem that is resistant to conventional medical intervention. Peripheral tissue damage and persistent inflammation are the major contributors to chronic pain (3). Clinically, patients with chronic pain often have emotional comorbidities, like anxiety and depression. The prevalence of anxiety disorders among patients with chronic pain ranges from 20% to 40% (4). In addition, anxiety often enhances patients’ suffering from pain, like for those affected by irritable bowel syndrome (IBS) (5). These studies suggested that chronic pain and anxiety may share similar targets and biological pathways, which affect concurrent treatments (6).

Several areas of the brain are involved in both chronic pain and anxiety, including the ACC, insular cortex (IC), and amygdala (7). Among these regions, the ACC is an ideal region to explore the integrations of pain and anxiety since it is a highly heterogeneous cortical region that includes both intrinsic and extrinsic connectivity (8). ACC neurons may receive nociceptive inputs from the thalamus and somatosensory cortices and emotional fear or anxiety information from the amygdala (7). Injuries were found to enhance synaptic transmission in the ACC. In addition, the inhibition of ACC potentiation produced analgesic and/or anxiolytic effects in animal models of chronic pain (9).

GIP, also known as gastric inhibitory polypeptide, is an endogenous peptide hormone synthesized and secreted by enteroendocrine K cells. GIP contains forty-two amino acids and is located primarily in the duodenum and proximal jejunum (10). GIP belongs to the incretin family, which potentiates insulin secretion after meal ingestion in a glucose-dependent manner. GIP and glucagon-like peptide-1 (GLP-1) are two crucial incretins that are highly expressed on islet β cells. They exert the insulinotropic actions through G-protein-coupled receptors (11). In addition to its effects on metabolism, some research has shown that GIP plays important roles in the central nervous system (CNS) as a type of vasoactive intestinal peptide (12, 13). GIP and its receptor, GIPR, are found in multiple brain regions. GIPR signaling may regulate synaptic plasticity, since acute intracerebroventricular injection of GIP promoted LTP in the hippocampus (14). Modulation of GIPR signaling provides a promising approach for the treatment of many diseases by suppressing neuronal viability decline, enabling neuronal regeneration, and reducing neuroinflammation (15). It has been demonstrated that activation of GIPR showed potential therapeutic effects in animal models for both Alzheimer’s disease (AD) (16) and Parkinson’s disease (PD) (17), which was closely related to reducing chronic inflammation response in the brain. These strongly suggest a potential role of GIPR in pain and anxiety modulation because neuroinflammation plays a key role in the maintenance of chronic pain and development of anxiety (18, 19).

In this study, the effects of GIPR activation and its potential mechanisms were investigated in mice with chronic inflammatory pain. The resulting data showed that activation of GIPR in the ACC attenuated pain and anxiety-like behaviors, potentially by inhibiting the excitatory synaptic transmission and inflammation in the ACC of mice models.

## Materials and Methods

### Animals

To exclude the influence of estrogen on pain perception in adult female mice, adult male C57BL/6 mice (8–12 weeks) from Laboratory Animal Center of the Fourth Military Medical University were used in the experiments. The mice were housed in plastic boxes with food and water available ad libitum in a colony room with controlled temperature (24 ± 2°C), humidity (50–60%), and a 12:12 h light-dark cycle. Animals were allowed to adapt to laboratory environment for at least one week prior to the beginning of experiments. Mice were randomly grouped. The number of experimental animals was reasonably minimized under the premise of satisfying the use and statistical analysis requirements. Experiments were performed under protocols approved by Animal Care and Use Committee of the Fourth Military Medical University. Complete Freund’s adjuvant (CFA) (10 μL, 50% in saline) was injected into the plantar surface of right hindpaw to induce chronic pain. The control mice received the same volume of saline.

### Materials

D-Ala^2^-GIP (purity ≥ 95%) and Pro^3^-GIP (purity ≥ 95%) were synthesized by GL Biochem Ltd (Shanghai, China). The purity of the peptide was analyzed by reversed-phase high performance liquid chromatography (HPLC) and characterized using matrix -assisted laser desorption/ionization time of flight (MALDI-TOF) mass spectrometry. D-Ala^2^-GIP sequence is Tyr-(D-Ala)-Glu-Gly-Thr-Phe-Ile-Ser-Asp-Tyr-Ser-Ile-Ala-Met-Asp-Lys-Ile-Arg-Gln-Gln-Asp-Phe-Val-Asn-Trp-Leu-Leu-Ala-Gln-Arg-Gly-Lys-Lys-Ser-Asp-Trp-Lys-His-Asn-Ile-Thr-Gln. Pro^3^-GIP sequence is Tyr-Ala-Pro-Gly-Thr-Phe-Ile-Ser-Asp-Tyr-Ser-Ile-Ala-Met-Asp-Lys-Ile-Arg-Gln-Gln-Asp-Phe-Val-Asn-Trp-Leu-Leu-Ala-Gln-Arg-Gly-Lys-Lys-Ser-Asp-Trp-Lys-His-Asn-Ile-Thr-Gln. Complete Freund’s adjuvant (CFA), Evans blue (E2129) and anti-β-actin antibody (A5316) were purchased from Sigma-Aldrich (Saint Louis, Missouri, USA). Anti-GluN2B (ab65783), anti-phosphorylated GluN2B at the S1303 site (p-GluN2B-S1303; ab81271), anti-GluA1 (ab31232), anti-Iba-1 (ab178847) and anti-p65 (ab16502) antibodies were purchased from Abcam (Cambridge, UK). Anti-phosphorylated GluA1 at the S845 site (p-GluA1-S845; ab5849) and anti-phosphorylated GluA1 at the S831 site (p-GluA1-S831; ab5847) antibodies were obtained from Millipore (Billerica, MA, USA). The following antibodies were purchased from Cell Signaling Technology (Danvers, MA, USA): glial fibrillary acidic protein (GFAP; #3670), anti-phosphorylated GluN2B at the T1472 site (p-GluN2B-T1472; #4208s), anti-cAMP-response element binding protein (CREB; #9197) and anti-phosphorylated CREB (p-CREB; #9198). HRP-conjugated anti-rabbit or anti-mouse secondary antibodies were purchased from Santa Cruz (CA, USA). Anti-GIPR antibody (abs122477) was purchased from Absin (Shanghai, China). TNF-α (VAL609), IL-1β (VAL601), and IL-6 (VAL604) Valukine ELISA kits were purchased from R&D Systems (Minnesota, USA). All other chemicals and reagents in this research are commercially available and standard in biochemical quality. GIPR adeno-associated virus (AAV) expressing EGFP and encoding short hairpin RNA (shRNA) was ordered from OBiO Technology (Shanghai, China).

### Mechanical Allodynia Test

Mice were put in individual plexiglas cages with a metal mesh floor and allowed to adjust to the environment for 30 min before testing. Mechanical allodynia was detected with a set of von Frey filaments (Aesthesio) and evaluated by hindpaw responsiveness to different stimulation (0.008–2 g). The hard lumps were not pricked to avoid false positives. By using Dixon’s up-down paradigm, the sensitivity of mechanical allodynia was determined. The filaments were applied vertically to the dorsal surface of the hindpaw to cause slight bending for 6 s. Positive responses included licking, biting, flinching and brisk withdrawal of the hindpaw. A rest interval of at least three minutes was allowed between consecutive stimulations. The results were tabulated and the pain threshold was assigned at 50% withdrawal.

### Thermal Hyperalgesia Test

Mice were placed in individual round container and allowed to adapt for 30 min before testing. A commercially available plantar analgesia instrument (PL-200, Chengdu Techman Software Co.,ltd., Chengdu, China) was used to detect the thermal nociceptive responses. Thermal hyperalgesia was determined by measuring the paw withdrawal latency (PWL) in response to a radiant heat source. The heat source was turned off when the mice lifted the foot, and PWL was defined as the time from beginning to the end of heat application. It was set maximum at 40 s to cut off the heat source automatically in order to prevent tissue damage even the mice did not lift foot. Paws were tested at 5 min intervals for a total of five trials and the mean PWL was calculated from the last three stimuli (20).

### Elevated Plus Maze Test

The elevated plus maze (EPM) test was performed as described in the previous report (21). The maze comprised of two open arms (25 cm × 8 cm × 0.5 cm) and two closed arms (25 cm × 8 cm × 12 cm) that extended from a central platform (8 cm × 8 cm). The arms were at a height of 50 cm above the floor. Mice were pretreated with gentle handling by experimenter twice per day for 7 days to minimize the nervousness. For each test, individual mice were placed in the center square, facing an open arm, and allowed to move freely for 5 min. Mice were videotaped using a camera fixed above the maze and analyzed with a video-tracking system (DigBehv-LR4, Shanghai Jiliang, Shanghai, China). The number of entries and time spent in each arm were recorded and calculated.

### Open Field Test

The open field (OF) test was performed in a square arena (30 cm × 30 cm × 30 cm) with clear Plexiglas walls and floor. It was placed inside an isolation soundproof box with dim illumination. Mice were placed in the center of the chamber and allowed to freely explore for 15 min. The ‘‘center’’ area was defined as the central 15 cm × 15 cm region, a quarter of the total area. A camera fixed above the floor recorded the action tracks of each mouse and a video-tracking system (DigBehv-LR4, Shanghai Jiliang, China) was used to analyze the results.

### Surgery and ACC Microinjection

Mice were anesthetized with 2.5% isoflurane (JiuPai, Shijiazhuang, China) in 100% oxygen with a rate of 0.5 L/min until loss of righting reflex, and maintained with 1.5% isoflurane in 100% oxygen with a rate of 0.5 L/min delivered by facemask. Mice were fixed in a stereotaxic frame (RWD68001, Shenzhen RWD Life Science, Shenzhen, China). A double cannula (guide cannula, length 9.5 mm, internal diameter 0.34 mm, external diameter 0.48 mm, center to center distance 0.6 mm) was aimed at 0.5 mm above the intended sites of injection, namely the ACC ( ± 0.03 cm lateral from the midline, +0.1 cm anterior to the bregma and -0.14 cm beneath from the surface of skull). The guide cannulas were affixed to the skull with machine screws and dental cement, and stylet was inserted into the cannula to keep them unimpeded. Mice were used for following experiments after one week recovery. To observe the injection location, 1% Evans blue (1 μL per site) was injected into the ACC through guide cannulas. For the injection of D-Ala^2^-GIP or Pro^3^-GIP, mice were anesthetized and fixed, then the drugs were bilaterally delivered at 0.5 μL/min using a syringe driven by an infusion pump (Harvard Apparatus, Inc., South Natick, USA). After that, the syringe was held in place for additional 1 min to permit the drug diffusion. For the injection of AAV, mice were anesthetized with isoflurane (3–4% for induced anesthesia and 1–1.5% for maintained anesthesia) and virus was microinjected into the ACC using a Hamilton syringe (10 μL) connected by glass capillary with tip (0.25μL/min for total 1μL per side) after skull drilling. The shRNA sequence for GIPR is 5’-GCTGCACTGCACTCGTAATTA-3’. AAV containing a nonsense control sequence (negative: CCTAAGGTTAAGTCGCCCTCG) was injected as negative control. The wounds were daubed with 0.5% iodophor to prevent infection after suture. To confirm the injection site of ACC, the brains were fixed with 4% paraformaldehyde and dehydrated through an ascending sucrose series. Then, they were sliced by a freezing microtome (Leica, Nussloch, Germany), and coronal sections (25 μm) containing the ACC were collected. The slices were mounted on glass slides and images were captured using a fluorescence microscope (Eclipse TE2000U; Nikon, Japan).

### Enzyme Linked Immuno-Sorbent Assay (ELISA) and Glucose Test

The levels of IL-1β, IL-6 and TNF-α in the serum were detected with ELISA kit for mouse (R&D Systems Inc., Minnesota, USA) following the instruction of manufacture. Briefly, the mice were anesthetized with diethyl ether. The eyeballs of mice were removed by ophthalmological forceps to break the blood vessels of fundus oculi. Blood (about 0.5 mL/mouse) was collected in the 1.5 mL centrifuge tube. The whole procedure was completed within 1 min. Subsequently, blood was centrifuged (1000 g) at 4°C for 20 min and the supernatant was collected for ELISA detection. For the detection of blood glucose level, the glucose test strips (Roche Diagnostics, Shanghai, China) were used following the instruction.

### Western-Blot Analysis

Western-blot analysis was conducted as previously described (22). Briefly, tissue samples from different brain areas were dissected carefully under the anatomical microscope and then dissociated with sonication in RIPA lysis buffer containing phosphatase inhibitor and protease inhibitor. Equal amounts of protein (30 μg) were separated with sodium dodecyl sulfate polyacrylamide gel and electro-transferred onto PVDF membranes (Invitrogen, Thermo Fisher Scientific Inc., Waltham, USA), which were probed with primary antibodies against β-actin (1:10000), GluN2B (1:1000), p-GluN2B-S1303 (1:1000), p-GluN2B-T1472 (1:1000), GluA1 (1:1000), p-GluA1 -S831 (1:1000), p-GluA1-S845 (1:1000), p-CREB (1:1000), CREB (1:1000), p65 (1:1000), Iba-1 (1:1000), GFAP (1:1000), and GIPR (1:500) overnight at 4°C. Subsequently, the membranes were incubated with anti-rabbit/anti-mouse horseradish peroxidase -conjugated secondary antibodies at room temperature for 1 h. The densitometric analysis of the Western-blot was performed with a ChemiDoc XRS (Bio-Rad, Hercules, CA, USA) and quantified with Image J software (NIH, Bethesda, Maryland) according to the instructions. Band intensity of each blot was calculated as ratio relative to β-actin. The intensity ratio of the control group was set as 100%, and the intensity of other groups was presented as percentage of control group.

### Immunofluorescence Staining

Immunofluorescence staining was performed according to our previous study (23). Mice were anesthetized with isoflurane in air and perfused with sterile saline, followed by 4% paraformaldehyde. Brains were removed and post-fixed in 4% paraformaldehyde overnight at 4°C. Free-floating coronal sections (10 μm) were obtained using a freezing microtome (Cryotome E, Thermo). Cortical sections containing the ACC were washed in 0.1 mM PBS buffer and permeabilized with 0.3% Triton in 5% normal goat serum for 1 h. Then sections were incubated in primary antibodies (Iba-1, 1:200; GFAP, 1:400) overnight at 4°C in 10% normal goat serum. After washing, sections were incubated with secondary Cy3-conjugated goat anti-mouse antibody (1:200; GB21301, Servicebio) or Alexa Fluor^®^ 488-conjugated goat anti-rabbit antibody (1:200; GB25303, Servicebio) for 2 hours at room temperature. Diluted DAPI in 0.1 mM PBS (1:1000) was applied to sections after washing to stain nuclei. The sections were mounted onto glass slides with cover glass using 50% glycerinum. For microglia/astrocyte morphology analysis, a stack of images spanning 5 μm thickness of the middle slice (1 μm thick z stacks) from Iba-1/GFAP-staining ACC slices were acquired using a confocal laser-scanning microscope. For the calculation of the cell-body area, the measurements were performed using the freehand selection tool in ImageJ. Sholl analysis was applied to analyze the branches and processes. Briefly, cells were cropped and thresholded to generate a binary (black and white) image. The background was manually cleaned for each cell and the ImageJ plugin ShollAnalysis was used to perform the analysis (starting radius: 4 μm, ending radius: 50 μm, radius step size: 2 μm).

### Quantitative Real-Time PCR (qRT-PCR)

Mice were deeply anesthetized with isoflurane and decapitated. The ACC was removed immediately. Total RNA was isolated using the mirVana RNA Isolation Kit (Life Technologies). The Goldenstar™ RT6 cDNA Synthesis Kit Ver 2 (Tsingke) was used to synthesize cDNA from mRNA. The reactions were performed using 2×T5 Fast qPCR Mix (SYBR Green I) (Tsingke). The sequences of primers were shown in **Supplementary Table 1**. The relative ratio of mRNA for each sample was calculated from the threshold cycles using a software program (StepOne, ABI) according to the manufacturer’s instructions. The expression levels of genes were normalized to *β-actin* expression.

### Whole-Cell Patch-Clamp Recording

Mice were anesthetized with isoflurane in air and then decapitated. Brains were rapidly removed and placed for 2-3 min in ice-cold and oxygenated artificial cerebrospinal fluid (ACSF) containing (in mM): 124 NaCl, 25 NaHCO_3_, 2.5 KCl, 1 KH_2_PO_4_, 2 CaCl_2_, 2 MgSO_4_ and 10 glucose, and continuously gassed with 95% O_2_/5% CO_2_. Coronal slices (350 μm) containing the ACC were prepared on a vibratome (Leica VT1200S) in ice-cold ACSF. Slices were then incubated in a room temperature-submerged recovery chamber with oxygenated (95% O_2_ and 5% CO_2_) ACSF for at least 1 h. After recovery, slices were transferred into a recording chamber on the stage of an Olympus microscope with infrared digital interference contrast optics for visualizing whole-cell patch-clamp recordings. Recordings were performed at room temperature (25 ± 1°C) with continuous perfusion of ACSF at a rate of 3 mL/min. For the recording of miniature excitatory postsynaptic currents (mEPSCs), recording pipettes (3-5 MΩ) were filled with solution containing 145 mM K-gluconate, 5 mM NaCl, 1 mM MgCl_2_, 0.2 mM EGTA, 10 mM HEPES, 2 mM Mg-ATP, and 0.1 mM Na_3_-GTP, adjusted to pH 7.2 with KOH (290 mOsm). Miniature EPSCs were recorded in the neurons clamped at −70 mV with the picrotoxin (100 μM) and tetrodotoxin (1 μM) in the ACSF, and they were analyzed using an event detection program (Mini Analysis Program; Synaptosoft, Inc., Decatur, GA). For the recording of paired-pulse ratio (PPR), a paired pulse paradigm was employed in which two stimuli were delivered at 50 ms inter-stimulus-interval (24). The stimulation electrode was placed in layer V/VI of the ACC. The ratio of the amplitude of the second response to the amplitude of the first response was calculated and averaged. The action potentials (APs) were recorded using the current-clamp mode in response to step currents injection. Depolarizing currents of 0–100 pA (400 ms duration) were delivered in increments of 10 pA. Access resistance (5-30 MΩ) was monitored throughout the experiment. Data were discarded if access resistance changed >15% during an experiment. All data were filtered at 1 kHz, and digitized at 10 kHz.

To identify the recorded neurons, biocytin (#B4261, Sigma-Aldrich, Saint Louis, Missouri, USA) was introduced into the recording solution with 0.4% final concentration. After recording, brain slices were immediately fixed in 4% paraformaldehyde overnight at 4°C. The sections were washed in 0.1 mM PBS buffer and permeabilized with 0.3% Triton in 5% normal goat serum for 1 h. After thoroughly washing with PBS, sections were incubated with Alexa Fluor^®^ 488-conjugated Streptavidin (1:500, #35103ES60, Yeasen Biotechnology (Shanghai) Co., Ltd. China) diluted in PBS overnight at 4°C. After washing, the slices were immediately mounted onto glass slides with cover glass using 50% glycerin and observed with a confocal microscope (FV-1000) under the appropriate filters for Alexa Fluor^®^ 488.

### Statistical Analysis

All behavioral, electrophysiological and biochemical analyses were performed blinded by the operators. All data were expressed as mean ± SEM. Comparison between two groups was analyzed by independent sample t-test. Data of multiple groups were evaluated using one-way analysis of variance (ANOVA) followed by Tukey test for *post hoc* comparisons or repeated-measure two-way ANOVA followed by LSD test for *post hoc* comparisons. Statistical analyses of the data were performed using SPSS 19.0. In all cases, *P* < 0.05 was considered as statistically significant.

## Results

### The Up-Regulation of GIPR in the ACC of Mice With CFA Injection

**Figure 1:**
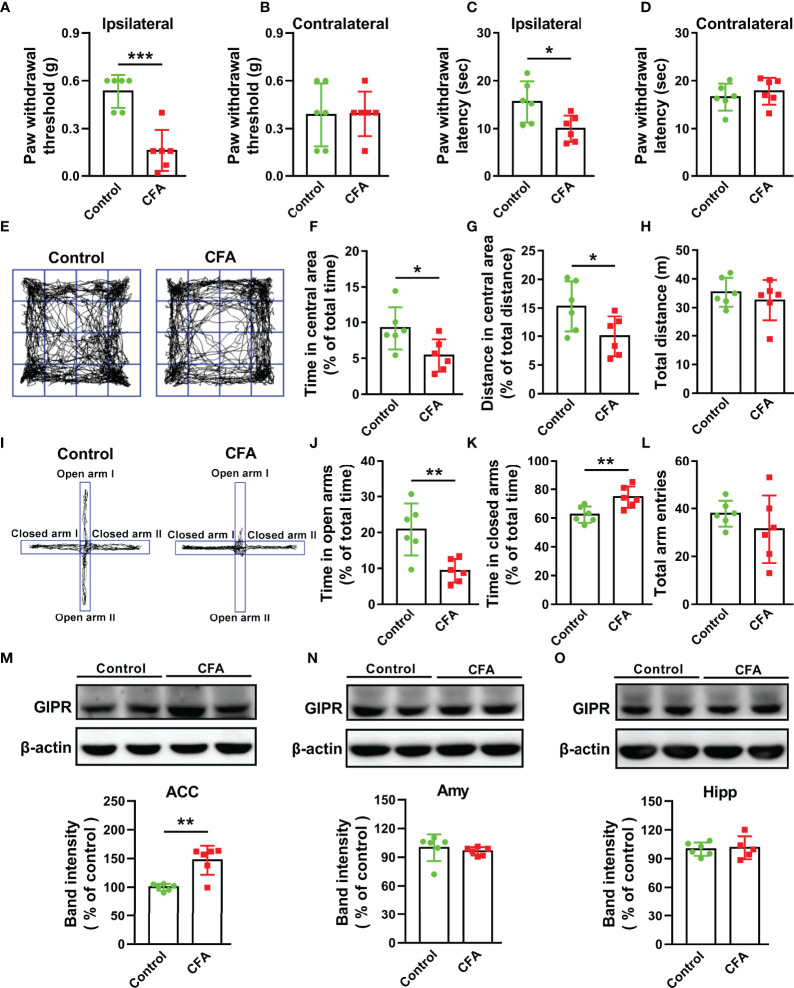
CFA injection induced nociceptive and anxiety-like behaviors along with the up-regulation of GIPR in the ACC. Paw withdrawal threshold and latency were decreased in CFA-injected paw (ipsilateral) **(A, C)** but not in non-CFA-injected paw (contralateral) **(B, D)**. **(E)** Representative traces in OF test. The time spent **(F)** and distance travelled **(G)** in the central area but not the total traveled distance **(H)** were decreased in mice injected with CFA. **(I)** Representative traces in EPM test. CFA-injected mice spent less time in open arms **(J)** and more time in closed arms **(K)** in EPM tests, but had similar total arm entries in open and close arms **(L)**. In CFA-treated mice, GIPR expression significantly increased in the ACC **(M)**, but not in the amygdala **(N)** and hippocampus **(O)**. n = 6 in each group except for CFA group of **Figure 10** (n = 5). Totally, 12 mice were used in this section. Amy means amygdala; Hipp means hippocampus. ^*^ *p* < 0.05, ^**^ *p* < 0.01, ^***^ *p* < 0.001.

Hind paw CFA injection is typically used to induce chronic inflammatory pain, which can last longer than two weeks (25). In this study, paw withdrawal threshold (T = 5.468, *P* = 0.0003, **Figures 1A, B**) and latency (T = 2.683, *P* = 0.023, **Figures C, D**) were significantly reduced in the CFA-injected paw (ipsilateral) as compared to the control on day 15 after injection. This suggests CFA-induced mechanical allodynia and thermal hyperalgesia. Chronic pain is an important incentive for negative emotions and they may share the same biological pathways and neurotransmitters (6). Anxiety-like behaviors were observed in mice with CFA injection. In the open field (OF) test, CFA significantly reduced the time spent and the distance travelled in the central area (T = 2.511, *P* = 0.0309, **Figures 1E, F**; T = 2.291, *P* = 0.045, **Figure 1G**), but no difference was observed in the total travelled distance (T = 0.7982, *P* = 0.4433, **Figure 1H**). In elevated plus maze (EPM) test, CFA markedly decreased the time spent in open arms (T = 3.546, *P* = 0.0053, **Figures 1I, J**) and increased the time spent in closed arms (T = 3.221, *P* = 0.0092, **Figure 1K**). However, the total arm entries showed no difference between the two groups (T = 1.054, *P* = 0.3165, **Figure 1L**). This indicates that CFA did not affect the normal locomotor activity in mice. These data suggest that CFA injection induces pain and anxiety-like behaviors in mice.

The limbic system of brain plays a key role in the regulation of pain perception and the related emotional hang-ups (7). In order to investigate whether GIPR was related to CFA-induced chronic pain and anxiety-like behaviors, Western blot analysis was done to confirm the expression and protein molecular weight of GIPR in the ACC, hippocampus, and amygdala (**Supplementary Figure 1**). CFA injection caused an increase in the level of GIPR in the ACC (T = 4.462, *P* = 0.0012, **Figure 1M**), but showed no difference in GIPR levels of the amygdala (T = 0.6274, *P* = 0.5445, **Figure 1N**) and hippocampus (T = 0.2415, *P* = 0.8146, **Figure 1O**). Because of this, we speculated that the GIPR in the ACC may be involved in the regulation of chronic pain and pain-related anxiety.

### The Analgesic and Anxiolytic Effects of GIPR Activation

**Figure 2:**
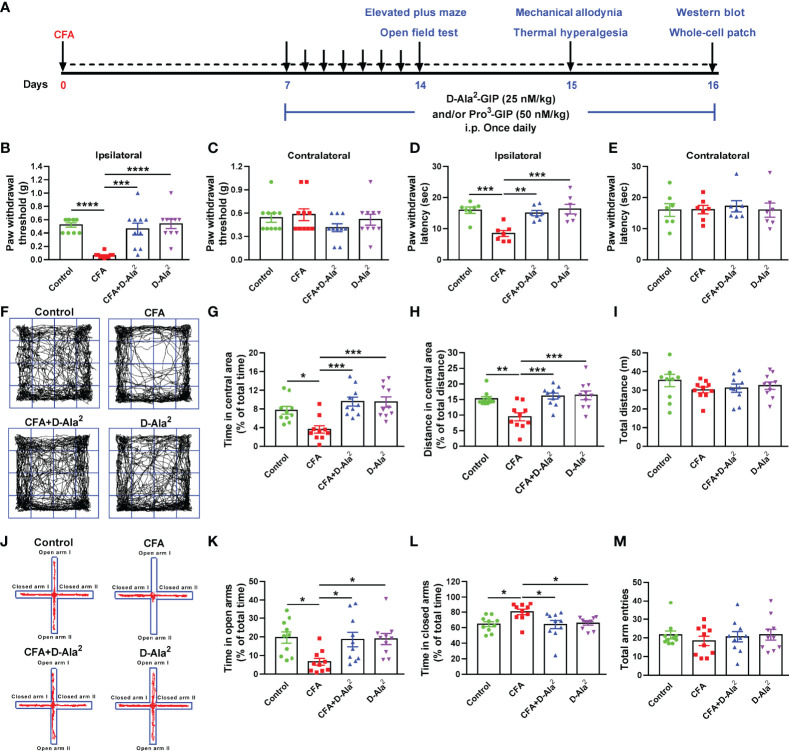
Systemic administration of GIPR agonist D-Ala^2^-GIP produced analgesic and anxiolytic effects. **(A)** The schedule of the experimental design. Systematic administration of D-Ala^2^-GIP (25 nM/kg, once daily for 10 days) increased the paw withdrawal threshold and latency in ipsilateral paw **(B, D)**, but did not affect those in contralateral paw **(C, E)**. **(F)** Representative traces in OF test. In OF test, D-Ala^2^-GIP treatment evidently increased the time spent **(G)** and distance travelled **(H)** in central area in CFA-injected mice, but had no effect on the total distance travelled **(I)**. **(J)** Representative traces in EPM test. In EPM test, systematic administration of D-Ala^2^-GIP (25 nM/kg, once daily for 10 days) increased the time spent in open arms **(K)**, decreased the time spent in closed arms **(L)** and did not affect the total arm entries **(M)**. n = 10 in each group except for the groups of **(D, E)** (n = 7). Totally, 68 mice were used in this section. D-Ala^2^ means D-Ala^2^-GIP. ^*^ *p* < 0.05, ^**^ *p* < 0.01, ^***^ *p* < 0.001, ^****^ *p* < 0.0001.

In order to determine the role of GIPR in CFA-induced chronic pain and anxiety-like behaviors, GIPR agonist D-Ala^2^-GIP was injected intraperitoneally (i.p.) at a dose of 25 nM/kg, once daily for 10 days. Equal volume of saline was injected intraperitoneally into the control and CFA mice. Treatment began on Day 7 after CFA injection. The anxiety-like behaviors and pain threshold of mice were detected on Days 14 and 15, respectively (22, 26) (**Figure 2A**). CFA injection was found to induce significant hyperalgesia in the ipsilateral paw (F_3,36_ = 14.43, *P* < 0.0001, **Figure 2B**; F_3,24_ = 10.71, *P* = 0.0005, **Figure 2D**), but not in the contralateral paw (non-CFA injection) (F_3,36_ = 1.22, *P* = 0.9719, **Figure 2C**; F_3,24_ = 0.09351, *P* > 0.9999, **Figure 2E**). D-Ala^2^-GIP showed evident analgesic effects demonstrated by the remarkable increase in the paw withdraw threshold, latency in mechanical allodynia test (F_3, 36_ = 14.43, *P* = 0.0002, **Figure 2B**), and withdraw latency in thermal test (F_3,24_ = 10.71, *P* = 0.0021, **Figure 2D**). In OF test, D-Ala^2^-GIP administration significantly enhanced the time spent (F_3,36_ = 9.013, *P* = 0.0003, **Figure 2G**) and the distance travelled in central area (F_3,36_ = 8.347, *P* = 0.001, **Figure 2H**) for CFA-injected mice. In EPM test, D-Ala^2^-GIP substantially increased the time spent in open arms (F_3,36_ = 4.256, *P* = 0.0385, **Figure 2K**) and decreased time spent in closed arms (F_3,36_ = 4.286, *P* = 0.0209, **Figure 2L**) for CFA-injected mice. D-Ala^2^-GIP had no effect on the total travelled distance in OF test (F_3,36_ = 0.8761, *P* = 0.9926, **Figure 2I**) or the total arm entries in EPM test (F_3,36_ = 0.3566, *P* = 0.9295, **Figure 2M**). It’s worth noting that the degree of pain and anxiety was almost restored to normal levels, which indicates that GIPR activation exerts excellent analgesic and anxiolytic effects.

**Figure 3:**
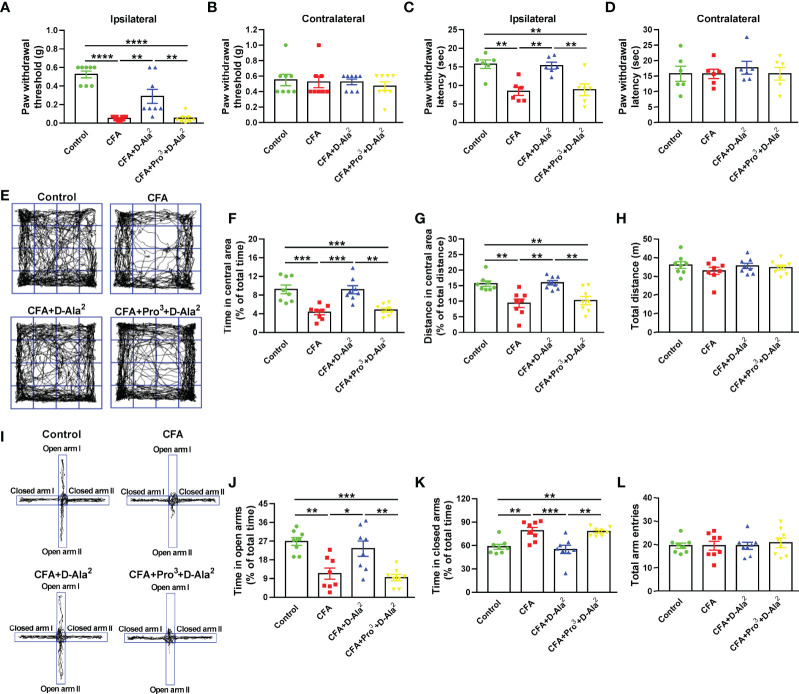
Pro^3^-GIP blocked the analgesic and anxiolytic effects of D-Ala^2^-GIP. Administration of Pro^3^-GIP prevented the D-Ala^2^-GIP-induced increases of paw withdrawal threshold and latency in CFA-injected mice **(A–D)**. Pro^3^-GIP also inhibited the anxiolytic effects of D-Ala^2^-GIP in OF test **(E–H)** and EPM test **(I–L)**. n = 8 in each group except for the groups of **(C, D)** (n = 6). Totally, 56 mice were used in this section. D-Ala^2^ means D-Ala^2^-GIP; Pro^3^ means Pro^3^-GIP. ^*^ *p* < 0.05, ^**^ *p* < 0.01, ^***^ *p* < 0.001, ^****^ *p* < 0.0001.

In order to further confirm the regulation of GIPR on pain and anxiety, GIPR antagonist Pro^3^-GIP was simultaneous intraperitoneally injected at a dose of 50 nM/kg once daily for 10 days. This was done along with the administration of D-Ala^2^-GIP (**Figure 2A**). We found that Pro^3^-GIP abolished the analgesic effects of D-Ala^2^-GIP, which was shown by the decreased ipsilateral paw withdrawal threshold (F_3,28_ = 27.68, *P* = 0.0032, **Figures 3A, B**) and latency (F_3,20_ = 10.49, *P* = 0.0065, **Figures 3C, D**). Similarly, Pro^3^-GIP also blocked the anxiolytic effects of D-Ala^2^-GIP in the OF test (F_3,28_ = 13.64, *P* = 0.0012, **Figures 3E, F**; F_3,28_ = 10.12, *P* = 0.0049, **Figures 3G, H**) and in the EPM test (F_3,28_ = 11.18, *P* = 0.0037, **Figures 3I, J**; F_3,28_ = 10.71, *P* = 0.0014, **Figures 3K, L**). Pro^3^-GIP alone had no effect on pain threshold and anxiety-like behaviors (**Supplementary Figure 2**) in either control or CFA-injected mice. These data indicate that activation of GIPR exerted both analgesic and anxiolytic effects in the mice with chronic inflammatory pain.

### The Role of ACC GIPR in the Chronic Inflammatory Pain and Comorbidities

**Figure 4:**
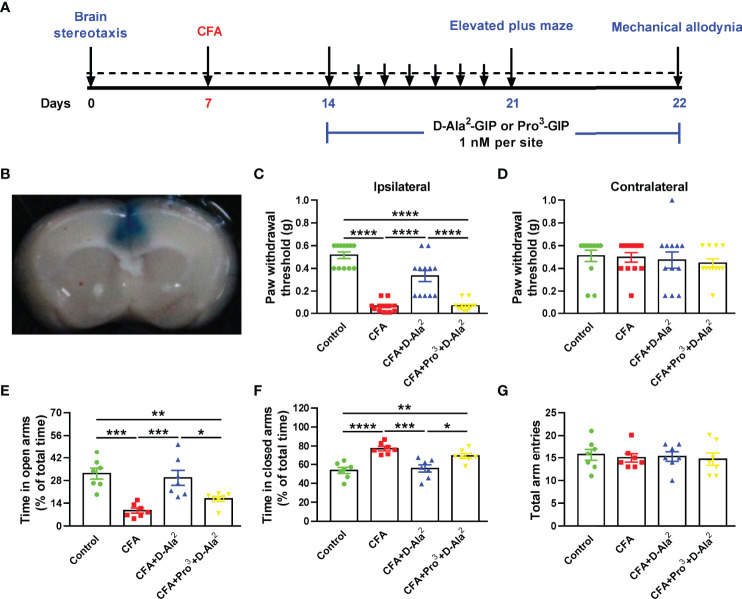
Local infusion of D-Ala^2^-GIP into the ACC also produced analgesic and anxiolytic effects. **(A)** Schedule showing the experimental design. **(B)** The injection location was shown by infusion of Evans blue. Local infusion of D-Ala^2^-GIP into the ACC increased the paw withdrawal threshold of ipsilateral paw, which was abolished by simultaneous infusion of Pro^3^-GIP **(C)**. Local infusion of D-Ala^2^-GIP and Pro^3^-GIP did not affect the contralateral paw **(D)**. In EPM test, local infusion of D-Ala^2^-GIP increased the time spent in open arms **(E)**, reduced the time spent in closed arms **(F)** and did not affect the total arm entries **(G)**. Simultaneous infusion of Pro^3^-GIP inhibited the effects of D-Ala^2^-GIP. n = 12 in each group except for the groups of **(E–G)** (n = 7). Totally, 48 mice were used in this section. D-Ala^2^ means D-Ala^2^-GIP; Pro^3^ means Pro^3^-GIP. ^*^ *p* < 0.05, ^**^ *p* < 0.01, ^***^ *p* < 0.001, ^****^ *p* < 0.0001.

In order to confirm the role of GIPR in the ACC, GIPR agonist or antagonist was microinjected into the ACC of CFA-injected mice, while saline was microinjected into the ACC of control and model mice (**Figures 4A, B**). D-Ala^2^-GIP microinjection (1 nM, 1 μL) also produced analgesic and anxiolytic effects, as shown by the increased paw withdrawal threshold in mechanical allodynia test (F_3, 44_ = 52.79, *P* < 0.0001, **Figures 4C, D**), the increased time spent in open arms (F_3, 24_ = 12.55, *P* = 0.0005, **Figure 4E**), and the decreased time spent in closed arms in EPM test (F_3, 24_ = 13.35, P = 0.0002, **Figures 4F, G**). Conversely, Pro^3^-GIP (1 nM, 1 μL) repressed the effects of D-Ala^2^-GIP (F_3, 44_ = 52.79, *P* < 0.0001, **Figure 4C**; F_3, 24_ = 12.55, *P* = 0.0287, **Figure 4E**; F_3, 24_ = 13.35, P = 0.0259, **Figure 4F**).

**Figure 5:**
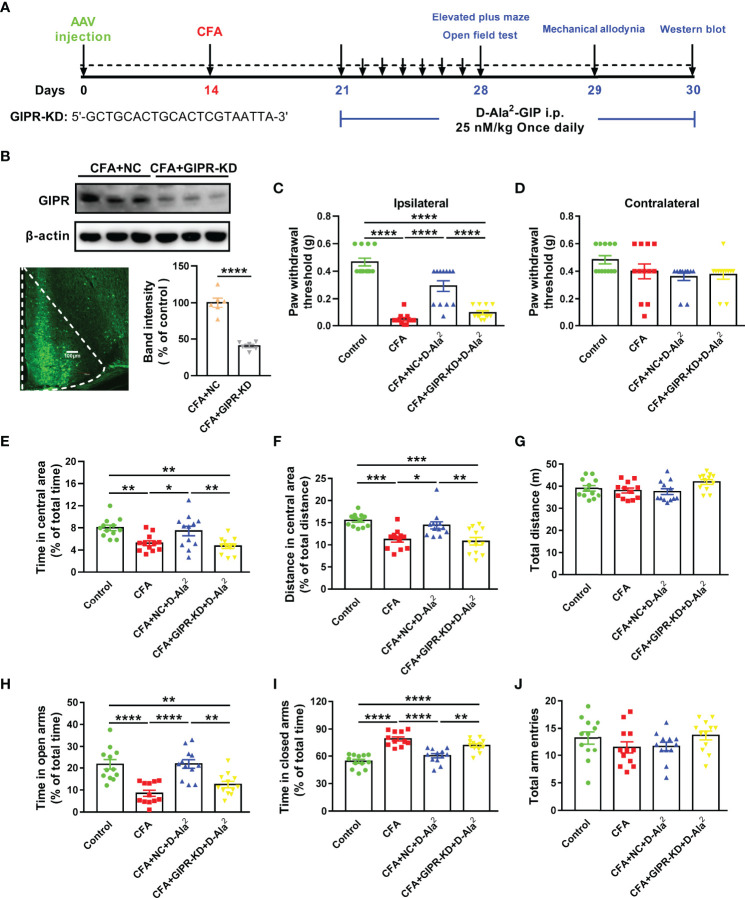
Knockdown of GIPR in the ACC blocked the analgesic and anxiolytic effects of D-Ala^2^-GIP. **(A)** The schedule of the experimental design. **(B)** The level of GIPR was knocked down in the ACC. GIPR shRNA transfection was shown by green fluorescent protein in coronal section. Scale bar: 100 μm. **(C, D)** GIPR shRNA transfection abolished the analgesic effect of D-Ala^2^-GIP. **(E–J)** GIPR knockdown abolished the effect of D-Ala^2^-GIP in OF test **(E–G)** and in EPM test **(H–J)**. n = 12 in each group except for the groups of **(B)** (n = 6). Totally, 48 mice were used in this section. NC means Negative Control; GIPR-KD means GIPR knockdown; D-Ala^2^ means D-Ala^2^-GIP; Pro^3^ means Pro^3^-GIP. ^*^ *p* < 0.05, ^**^ *p* < 0.01, ^***^ *p* < 0.001, ^****^ *p* < 0.0001.

To further prove the effect of GIPR in the ACC, we knocked down GIPR expression in the ACC with the use of shRNA transfection (**Figure 5A**). Adeno-Associated Virus (AAV) that carried specific GIPR shRNA was successfully microinjected into the ACC (**Figure 5B**). The protein level of GIPR in the ACC was reduced to 40.47 ± 2.27% of the negative control (T = 8.69, p < 0.0001, **Figure 5B**). In the mechanical allodynia test, GIPR shRNA transfection in the ACC eliminated the analgesic effects of D-Ala^2^-GIP *via* intraperitoneal injection in CFA-treated mice (F_3,44_ = 55.61, P < 0.0001, **Figures 5C, D**). The anxiolytic effect of D-Ala^2^-GIP was also inhibited by GIPR shRNA transfection in the OF test (F_3,44_ = 7.854, P = 0.0091, **Figure 5E**; F_3,44_ = 11.21, P = 0.0039, **Figures F, G**) and the EPM test (F_3,44_ = 14.28, P = 0.0029, **Figure 5H**; F_3,44_ = 27.21, P = 0.002, **Figures 5I, J**). These data suggest that ACC GIPR mediates analgesic and anxiolytic effects in mice with chronic inflammatory pain.

### No Peripheral Anti-Inflammatory Effects Were Observed After GIPR Activation

**Figure 6:**
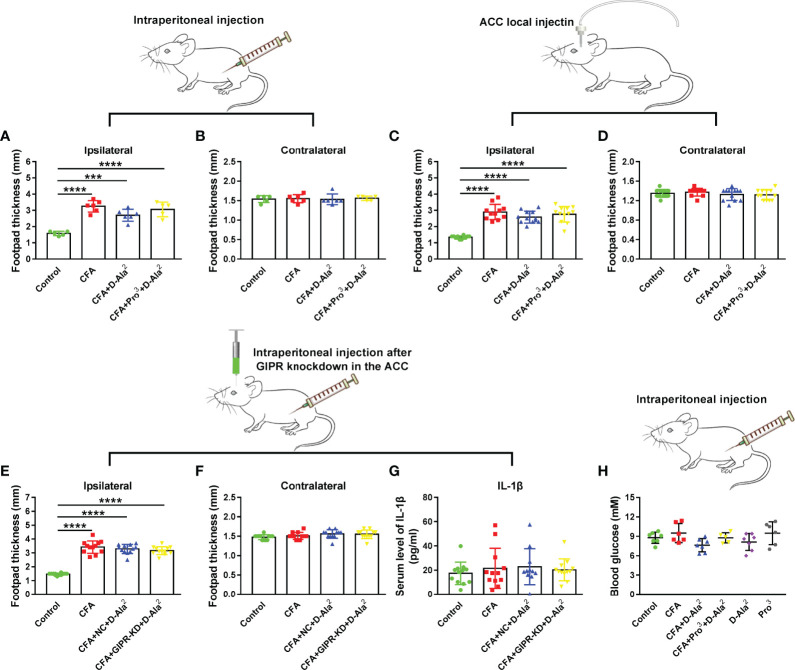
GIPR activation had no effect on peripheral inflammation and glucose level. Systematic administration of D-Ala^2^-GIP **(A, B)** (n = 5 in control and CFA+Pro^3^+D-Ala^2^ groups, n = 6 in CFA and CFA+D-Ala^2^ groups) or ACC local injection of D-Ala^2^-GIP **(C, D)** (n = 11 in CFA group, n = 12 in other groups**)** did not reduce edema in CFA-injected hindpaw. GIPR knockdown did not reduce the footpad thickness in CFA-injected hindpaws **(E, F)** (n = 12 per group). There was no distinguished difference in the level of serum IL-1β **(G)** (n = 11 in CFA+D-Ala^2^ group, n = 12 in other groups**)** among the groups. D-Ala^2^-GIP or Pro^3^-GIP had no evident effect on the blood glucose level **(H)** (n = 7 in control, CFA+D-Ala^2^ and D-Ala^2^ groups; n = 6 in CFA and Pro^3^ groups; n = 5 in CFA+Pro^3^+D-Ala^2^ group). Totally, 155 mice were used for statistics in this figure. NC means Negative Control; GIPR-KD means GIPR knockdown; D-Ala^2^ means D-Ala^2^-GIP; Pro^3^ means Pro^3^-GIP. ^***^ *p* < 0.001, ^****^ *p* < 0.0001.

Inflammation is an important contributor to chronic pain and emotional disorders (27). In order to verify whether the activation of GIPR had any peripheral anti-inflammatory effects, the footpad thickness and levels of inflammatory cytokines were measured. As shown in **Figure 6A**, CFA injection caused evident edema in the ipsilateral hind paw. Systemic intraperitoneal injection or ACC local infusion of D-Ala^2^-GIP had no effect on footpad thickness (F_3,18_ = 23.85, *P* = 0.0607, **Figure 6A**; F_3,43_ = 41.01, *P* = 0.2127, **Figure 6C**). Similarly, Pro^3^-GIP (**Figures 6A–D**) or GIPR knockdown (**Figures 6E, F**) also had no influence on footpad thickness. ELISA was used to detect the serum levels of inflammatory cytokines including IL-1β, IL-6, and TNF-α. The levels of IL-6 and TNF-α were below the detection limit. The activation of GIPR had no effect on the serum levels of IL-1β (F_3, 43_ = 0.3804, *P* = 0.7676, **Figure 6G**). These results suggest that the effects of GIPR activation were not due to the peripheral anti-inflammatory activities.

GIPR is a potential target for the treatment of type 2 diabetes mellitus (28). In order to investigate this, we tested the blood glucose of mice to exclude the possible influence of blood glucose level on their behaviors. The results showed that systemic administration of D-Ala^2^-GIP and/or Pro^3^-GIP once daily for 10 days did not affect blood glucose levels (F_5, 32_ = 2.305, *P* = 0.0675, **Figure 6H**). This suggests that the activation or inhibition of GIPR does not affect blood glucose levels in normal animals.

### GIPR Activation Attenuated Neuroinflammation in the ACC

**Figure 7:**
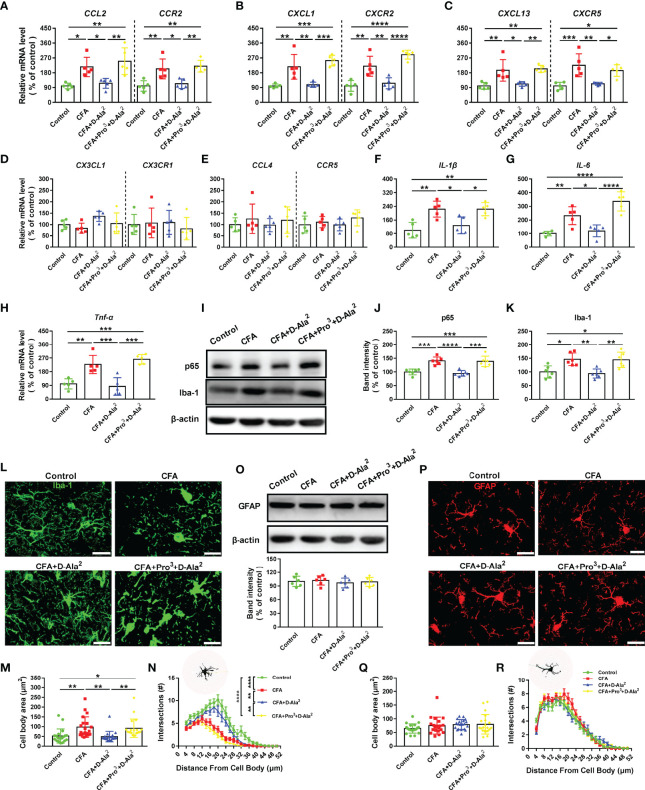
GIPR activation inhibited inflammation and microglia activation in the ACC. The mRNA levels of CCL2/CCR2 **(A)**, CXCL1/CXCR2 **(B)**, CXCL13/CXCR5 **(C)**, CX3CL1/CX3CR1 **(D)**, CCL4/CCR5 **(E),** IL-1β **(F)**, IL-6 **(G)** and TNF-α **(H)** were detected by qRT-PCR analysis in the ACC (n = 5 in each group). **(I)** Representative Western blot analysis of NF-κB p65 and Iba-1. **(J, K)** CFA injection significantly increased NF-κB p65 and Iba-1 levels in the ACC. D-Ala^2^-GIP (i.p. injection) notably reversed the increased expressions of NF-κB p65 and Iba-1, which was inhibited by Pro^3^-GIP (i.p. injection, n = 6 in each group). **(L–N)** Immunostaining of Iba-1 **(L)**, quantification of the microglial cell body area **(M)**, and Sholl analysis of microglial cells **(N)**. n = 20 cells from 3 mice per group. Scale bar: 20 μm. **(O–R)** The GFAP expression **(O)** and morphology of astrocyte **(P–R)** were unaffected in the ACC. n = 20 cells from 3 mice per group. Totally, 56 mice were used in this figure. Scale bar: 20 μm. D-Ala^2^ means D-Ala^2^-GIP; Pro^3^ means Pro^3^-GIP. ^*^ *p* < 0.05, ^**^ *p* < 0.01, ^***^ *p* < 0.001, ^****^ *p* < 0.0001.

Inflammation in the CNS is an important contributor to chronic pain and anxiety. First, we detected several chemokine/receptor pairs in the ACC: CCL2/CCR2, CXCL1/CXCR2, CXCL13/CXCR5, CX3CL1/CX3CR1, and CCL4/CCR5. These are reported to be associated with the pathogenesis of chronic inflammatory pain (29). The mRNA levels of *CCL2/CCR2*, *CXCL1/CXCR2*, and *CXCL13/CXCR5* were remarkably higher in CFA-injected mice than those of control (F_3, 16_ = 9.747, *P* = 0.0158, F_3, 16_ = 11.18, *P* = 0.0051, **Figure 7A**; F_3, 16_ = 16.89, *P* = 0.0024, F_3, 16_ = 24.47, *P* = 0.0013, **Figure 7B**; F_3, 16_ = 11.1, *P* = 0.0041, F_3, 16_ = 11.06, *P* = 0.0009, **Figure 7C**). D-Ala^2^-GIP i.p. treatment significantly reduced the levels of *CCL2/CCR2*, *CXCL1/CXCR2*, and *CXCL13/CXCR5* (F_3, 16_ = 9.747, *P* = 0.0402, F_3, 16_ = 11.18, *P* = 0.0146, **Figure 7A**; F_3, 16_ = 16.89, *P* = 0.0044, F_3, 16_ = 24.47, *P* = 0.0046, **Figure 7B**; F_3, 16_ = 11.1, *P* = 0.0117, F_3, 16_ = 11.06, *P* = 0.0027, **Figure 7C**), and this reduction was inhibited by Pro^3^-GIP i.p. treatment (F_3, 16_ = 9.747, *P* = 0.0048, F_3, 16_ = 11.18, *P* = 0.0041, **Figure 7A**; F_3, 16_ = 16.89, *P* = 0.0003, F_3, 16_ = 24.47, *P* < 0.0001, **Figure 7B**; F_3, 16_ = 11.1, *P* = 0.0051, F_3, 16_ = 11.06, *P* = 0.032, **Figure 7C**). *CX3CL1/CX3CR1* and *CCL4/CCR5* showed no difference among these groups (F_3, 20_ = 2.711, *P* = 0.0797, F_3, 20_ = 0.2612, *P* = 0.8522, **Figure 7D**; F_3, 20_ = 0.3951, *P* = 0.7583, F_3, 20_ = 1.042, *P* = 0.4008, **Figure 7E**). We also detected the mRNA expression of *IL-1β*, *IL-6*, and *TNF-α* in the ACC. The expressions of *IL-1β*, *IL-6*, and *TNF-α* in CFA-injected mice were higher than those in the control group (F_3, 16_ = 10.05, *P* = 0.0027, **Figure 7F**; F_3, 16_ = 21.01, *P* = 0.0062, **Figure 7G**; F_3, 16_ = 17.85, *P* = 0.003, **Figure 7H**). D-Ala^2^-GIP administration markedly decreased the expressions of *IL-1β*, *IL-6*, and *TNF-α* (F_3, 16_ = 10.05, *P* = 0.0199, **Figure 7F**; F_3, 16_ = 21.01, *P* = 0.0207, **Figure 7G**; F_3, 16_ = 17.85, *P* = 0.001, **Figure 7H**). These effects were also blocked by Pro^3^-GIP (F_3, 16_ = 10.05, *P* = 0.0199, **Figure 7F**; F_3, 16_ = 21.01, *P* < 0.0001, **Figure 7G**; F_3, 16_ = 17.85, *P* = 0.0001, **Figure 7H**).

The activation of microglia is required for the onset and progression of inflammation (30, 31).NF-κB p65, which is a protein complex that regulates immune response, and Iba-1, a microglia marker, were detected by Western blot analysis. The expressions of NF-κB p65 and Iba-1 were markedly increased in the ACC of CFA-injected mice (F_3, 20_ = 18.69, *P* = 0.0003, **Figures 7I, J**; F_3, 20_ = 9.029, *P* = 0.0106, **Figure 7K**). The up-regulations of NF-κB p65, and Iba-1 returned to base levels after D-Ala^2^-GIP treatment (F_3, 20_ = 18.69, *P* < 0.0001, **Figure 7J**; F_3, 20_ = 9.029, *P* = 0.004, **Figure 7K**) (also seen in **Supplementary Figures 3A, B, D**). These effects were blocked by Pro^3^-GIP (F_3, 20_ = 18.69, *P* = 0.0001, **Figure 7J**; F_3, 20_ = 9.029, *P* = 0.0056, **Figure 7K**). The morphology of microglia was analyzed by Iba-1 immunostaining. Microglia of CFA-injected mice presented with enlarged cell bodies and branching impairment (F_3, 76_ = 7.463, *P* = 0.0056, **Figure 7M, L, N**). D-Ala^2^-GIP treatment rescued this microglial morphology (F_3, 76_ = 7.463, *P* = 0.0024, **Figures 7M, L, N**), which could be reversed by Pro^3^-GIP (F_3, 76_ = 7.463, *P* = 0.0095, **Figures 7M, L, N**). There was no difference in GFAP expression, an astrocyte marker, among these groups (F_3, 20_ = 0.2862, *P* = 0.8348, **Figure 7O**; also seen in **Supplementary Figures 3A, C**). The morphology of astrocytes was also unchanged (F_3, 76_ = 1.4, *P* = 0.2493, **Figures 7Q, P, R**). These data implied that the analgesic and anxiolytic effects of GIPR were associated with inhibition of CNS inflammation.

### Effects of GIPR on Synaptic Proteins in the ACC

**Figure 8:**
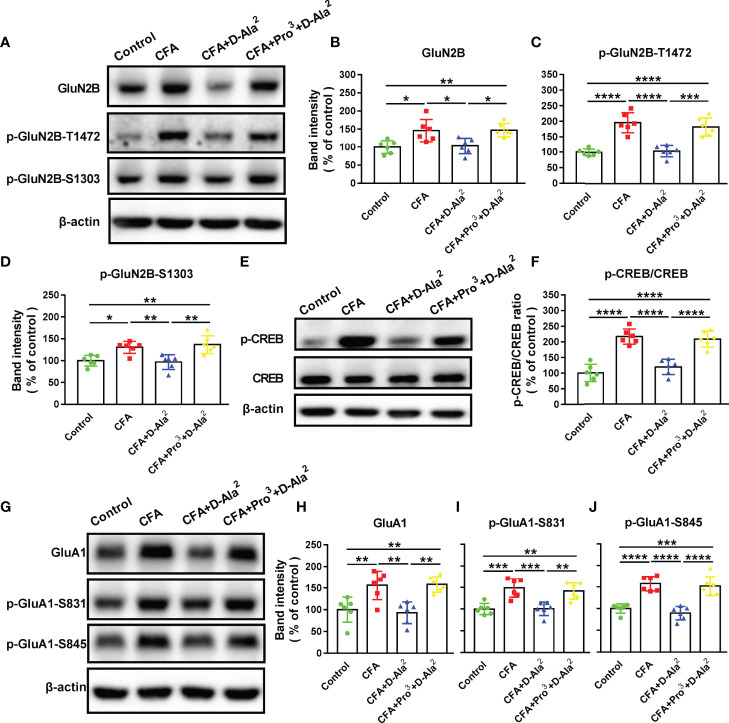
Effects of GIPR activation on the levels of synaptic plasticity-related proteins in the ACC. **(A)** Representative Western blot analysis of GluN2B, p-GluN2B-T1472 and p-GluN2B-S1303. **(B–D)** Systemic administration of D-Ala^2^-GIP reversed the up-regulation of above proteins in the ACC of model mice, and Pro^3^-GIP inhibited the effects of D-Ala^2^-GIP. **(E)** Representative Western blot analysis of p-CREB and CREB. **(F)** D-Ala^2^-GIP inhibited the increase of p-CREB/CREB ratio, which was abolished by Pro^3^-GIP. **(G)** Representative Western blot analysis of GluA1, p-GluA1-S831 and p-GluA1-S845. **(H–J)** D-Ala^2^-GIP treatment reversed the up-regulations of GluA1, p-GluA1-S831 and p-GluA1-S845 in the ACC of model mice, and Pro^3^-GIP blocked the effects of D-Ala^2^-GIP. n = 6 in each group. Totally, 24 mice were used in this section. D-Ala^2^ means D-Ala^2^-GIP; Pro^3^ means Pro^3^-GIP. ^*^ *p* < 0.05, ^**^ *p* < 0.01, ^***^ *p* < 0.001, ^****^ *p* < 0.0001.

Next, we examined the changes of glutamate receptor expression in the ACC, since glutamate N-methyl-D-aspartate (NMDA) receptors contributed to behavioral abnormalities induced by inflammation (32). Among the subunits of NMDA receptors, GluN2B is especially involved in CFA-induced inflammatory pain modulation in the ACC (33, 34), and the phosphorylation of GluN2B at T1472 (p-GluN2B-T1472) and S1303 (p-GluN2B-S1303) enhances GluN2B-NMDA receptor current (35–37). The levels of GluN2B, p-GluN2B-T1472 and p-GluN2B-S1303 were evidently increased in the ACC of CFA-injected mice as compared to the control (F_3, 20_ = 7.788, *P* = 0.0112, **Figures 8A, B**; F_3, 20_ = 26.21, *P* < 0.0001, **Figure 8C**; F_3, 20_ = 9.922, *P* = 0.0173, **Figure 8D**). D-Ala^2^-GIP i.p. treatment reversed the up-regulation of these proteins that was previously induced by CFA (F_3, 20_ = 7.788, *P* = 0.0193, **Figure 8B**; F_3, 20_ = 26.21, *P* < 0.0001, **Figure 8C**; F_3, 20_ = 9.922, *P* = 0.008, **Figure 8D**) (also seen in **Supplementary Figures 3E–H**). Simultaneous Pro^3^-GIP i.p. treatment inhibited the effects of D-Ala^2^-GIP (F_3, 20_ = 7.788, *P* = 0.0162, **Figure 8B**; F_3, 20_ = 26.21, *P* = 0.0001, **Figure 8C**; F_3, 20_ = 9.922, *P* = 0.0017, **Figure 8D**).

The cAMP-response element-binding protein (CREB) can be activated by the NMDA receptor (38). Accordingly, the levels of phosphorylated CREB (p-CREB) and total CREB were detected. To detect the activation degree of CREB signaling, the ratio of p-CREB/CREB was calculated and found to be significantly increased in the ACC of model mice as compared to the control (F_3, 20_ = 33.05, *P* < 0.0001, **Figures 8E, F**). This was reversed by D-Ala^2^-GIP treatment (F_3, 20_ = 33.05, *P* < 0.0001, **Figure 8F**) (also seen in **Supplementary Figures 3I, J**), while Pro^3^-GIP inhibited the effects of D-Ala^2^-GIP (F_3, 20_ = 33.05, *P* < 0.0001, **Figure 8F**). The activation of CREB leads to the phosphorylation of GluA1-containing α-amino-3-hydroxy-5-methyl-4-isoxazolepropionic acid (AMPA) receptor, which is another important glutamate receptor that is closely connected with the regulation of chronic pain and anxiety (39). The phosphorylation of GluA1 at S831 (p-GluA1-S831) and S845 (p-GluA1-S845) controls receptor expression and function (40). The total GluA1, p-GluA1-S831 and p-GluA1-S845 were found to be significantly up-regulated in the ACC of CFA-injected mice as compared to the control (F_3, 20_ = 10.35, *P* = 0.0084, **Figures 8G, H**; F_3, 20_ = 13.17, *P* = 0.0006, **Figure 8I**; F_3, 20_ = 25.69, *P* < 0.0001, **Figure 8J**). D-Ala^2^-GIP administration inhibited the up-regulation of GluA1, p-GluA1-S831, and p-GluA1-S845 in the ACC of model mice (F_3, 20_ = 10.35, *P* = 0.003, **Figure 8H**; F_3, 20_ = 13.17, *P* = 0.0007, **Figure 8I**; F_3, 20_ = 25.69, *P* < 0.0001, **Figure 8J**) (also seen in **Supplementary Figures 3K-N**). The effects of D-Ala^2^-GIP were abolished by Pro^3^-GIP treatment (F_3, 20_ = 10.35, *P* = 0.0022, **Figure 8H**; F_3, 20_ = 13.17, *P* = 0.0034, **Figure 8I**; F_3, 20_ = 25.69, *P* < 0.0001, **Figure 8J**). These results hinted that activation of GIPR may regulate excitatory neurotransmission in the ACC.

### Effects of GIPR Activation on Excitatory Synaptic Transmission in the ACC

**Figure 9:**
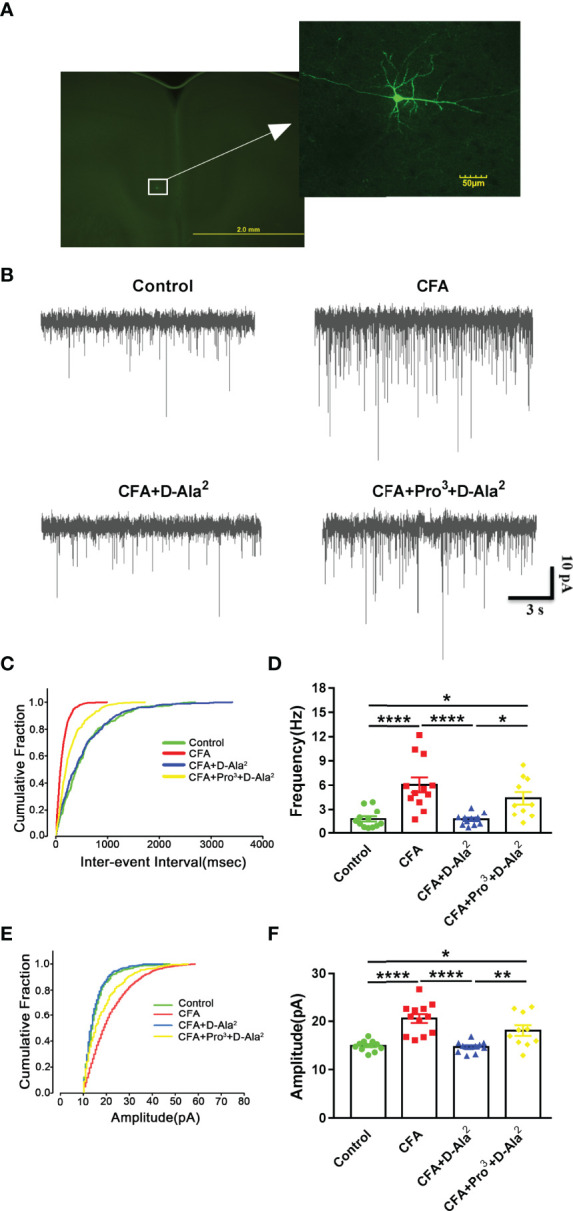
GIPR activation reduced the enhanced synaptic transmission in the ACC. **(A)** Representative picture showing the location of the recorded pyramidal neurons in the ACC. **(B)** Representative mEPSCs recorded in pyramidal neurons of ACC at a holding potential of −70 mV. **(C, D)** Cumulative inter-event interval plot of recorded mEPSCs and summary plots of mEPSC frequency (n = 12 in control, CFA and CFA+D-Ala^2^ groups; n = 10 in CFA+Pro^3^+D-Ala^2^ group). **(E, F)** Cumulative plot of mEPSCs amplitude and summary plots of mEPSC amplitude (n = 12 in control, CFA and CFA+D-Ala^2^ groups; n = 10 in CFA+Pro^3^+D-Ala^2^ group). The traces in cumulative fraction plot represent mean values of each group. Totally, 24 mice were used in this section. D-Ala^2^-GIP treatment reversed the enhancement of mEPSCs frequency and amplitude in CFA-treated mice, which was attenuated by Pro^3^-GIP. D-Ala^2^ means D-Ala^2^-GIP; Pro^3^ means Pro^3^-GIP. ^*^ *p* < 0.05, ^**^ *p* < 0.01, ^****^ *p* < 0.0001.

Chronic pain and the related emotional disorders are accompanied by an abnormal enhancement of excitatory neurotransmission in the limbic system (41). In order to assess the role of GIPR in the excitatory synaptic transmission in the ACC, whole cell patch-clamp recordings were performed. As layer II/III of ACC receive afferent pain signals and are main information processing layers, miniature excitatory postsynaptic currents (mEPSC) from pyramidal neurons in layer II/III of ACC were recorded on Day 16 after CFA injection (**Figures 2A**, **9A, B**). The results showed that the frequency and amplitude of mEPSCs were significantly increased in ACC neurons from model mice as compared to the control (F_3, 42_ = 12.12, *P* < 0.0001, **Figure 9D**; F_3, 42_ = 16.05, *P* < 0.0001, **Figure 9F**). The cumulative fraction plot showed a decreased inter-event-interval and increased amplitude in model mice as compared to the control (**Figures 9C, E**). D-Ala^2^-GIP i.p. treatment reduced the CFA-induced enhancement of mEPSCs (F_3, 42_ = 12.12, *P* < 0.0001, **Figure 9D**; F_3,42_ = 16.05, *P* < 0.0001, **Figure 9F**), which was abolished by Pro^3^-GIP i.p. treatment (F_3, 42_ = 12.12, *P* = 0.0186, **Figure 9D**; F_3, 42_ = 16.05, *P* = 0.0085, **Figure 9F**). These results were confirmed by corresponding alterations in the cumulative probabilities of mEPSC frequency and amplitude (**Figures 9C, E**).

**Figure 10:**
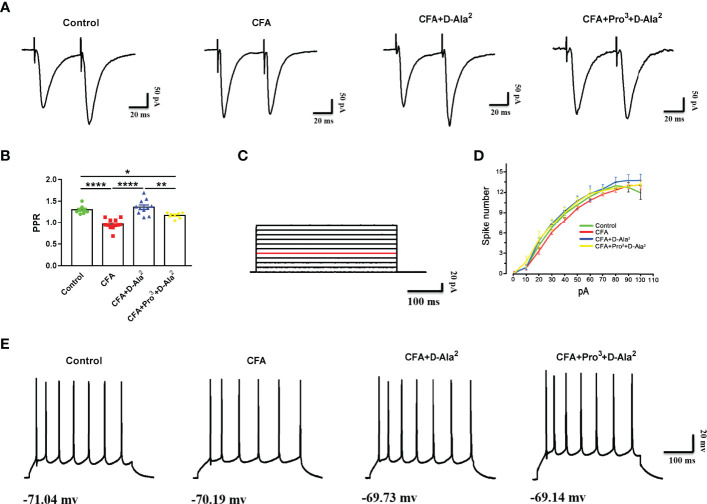
GIPR activation inhibited the reduction of PPR and had no effect on neuronal excitability. **(A)** Representative traces showing the paired-pulse ratio (PPR) recorded with a 50 ms interval. **(B)** D-Ala^2^-GIP inhibited the reduction of PPR in ACC neurons induced by CFA-injection (n = 8 in control group; n = 12 in CFA group; n = 11 in CFA+D-Ala^2^ group), and Pro^3^-GIP attenuated the effects of D-Ala^2^-GIP (n = 8). **(C)** Injection of step currents from 10 pA to 100 pA with 10 pA steps. **(D)** Curve of spike numbers of action potential induced by injected currents in ACC excitatory neurons. D-Ala^2^-GIP or Pro^3^-GIP had no effect on the firing rate of pyramidal neurons (n = 12 in control and CFA groups; n = 13 in CFA+D-Ala^2^ group; n = 11 in CFA+Pro^3^+D-Ala^2^ group). Totally, 24 mice were used in this section. **(E)** Representative traces (40 pA injection current) showing the firing property of the pyramidal neurons from ACC response to D-Ala^2^-GIP or Pro^3^-GIP. D-Ala^2^ means D-Ala^2^-GIP; Pro^3^ means Pro^3^-GIP. ^*^ *p* < 0.05, ^**^ *p* < 0.01, ^****^ *p* < 0.0001.

In order to investigate the role of presynaptic and/or postsynaptic mechanisms in excitatory synaptic transmission after GIPR activation, we examined paired-pulse ratio (PPR) in the ACC. We found that CFA induced the reduction of PPR at a stimulus interval of 50 milliseconds in ACC pyramidal neurons (F_3,35_ = 22.54, *P* < 0.0001, **Figure 10B**), indicating the enhancement of presynaptic neurotransmission. D-Ala^2^-GIP i.p. treatment suppressed the decreased PPR (F_3, 35_ = 22.54, *P* < 0.0001, **Figure 10B**), and the effects of D-Ala^2^-GIP could be inhibited by Pro^3^-GIP (F_3, 35_ = 22.54, *P* = 0.0024, **Figure 10B**). These data indicate that the analgesic and anxiolytic effects of GIPR are associated with the inhibition of presynaptic neurotransmission. Next, we examined biophysical characteristics of action potentials (APs) in order to investigate the effect of GIPR on neuronal excitability. APs were evoked by positive current injection in current-clamp model. We found that there was no significant difference in the spike numbers of APs at the same current injection among each group (**Figures 10C–E**). This finding indicated that GIPR activation did not affect the excitability of pyramidal neurons in the ACC.

## Discussion

Chronic pain is a powerful motivator that is able to induce emotional disorders, like anxiety and depression (42, 43). Conversely, anxiety can lead to persistent and refractory chronic pain (44). The positive interaction between chronic pain and anxiety suggests that they may share some similar biological pathways and neurotransmitters. Chronic inflammatory pain is an ideal model to explore these mechanisms since it is a common type of pain. In rodents, CFA-induced inflammatory pain usually persists from weeks to months, so its pathophysiological features are suitable for studying chronic inflammatory pain and pain-related psychiatric comorbidities (45). Although previous studies reported that mice injected with CFA did not show anxiety-like behaviors (46), animal behaviors may be influenced by different injection methods, experimental protocols, animal species, and environmental factors. The results from this study were consistent with many other reports and our previous work (47, 48). Our results showed that intraplanar injection of CFA induced obvious hyperalgesia and anxiety-like behaviors.

As an important incretin, the peripheral roles of GIP in metabolism, diabetes, obesity, appetite control, and other physiological functions have been well documented in previous research (11, 49). In the CNS, GIPR is expressed not only in neurons but also in microglia, astrocytes, and oligodendrocytes (50, 51). The nonspecific expression of GIPR suggests that it has multiple roles in the CNS. Our data showed that GIPR activation inhibited the neuroinflammation as well as reduced excessive excitatory transmission in synapses. As neurons and glia cells interact with each other, we infer that neuronal and glial GIPRs play important roles in analgesia and anti-anxiety in the ACC. It has been reported that activation of GIPR prevented learning and memory deficits by inhibiting beta amyloid plaque deposition and neuroinflammation in AD animal models (16). GIPR activation also promoted the survival of dopaminergic neurons in the substantia nigra *pars compacta* (SNpc). It also improved motor activity in PD animal models (17, 49). Recent literature revealed that activation of GIPR in the CNS significantly decreased body weight and blood glucose in mice fed with a high-fat diet (HFD) or mice with diet-induced obesity (DIO) (52).

In the present study, the ad libitum plasma levels of blood glucose in mice were unchanged after GIPR agonist and/or antagonist treatment. This is consistent with the results in healthy humans (53). This seemingly conflicting observation could be attributed to the glucose-dependent insulin tropic effects of GIP. HFD or DIO mice often have an abnormal glucose metabolism as well; however, the mice used in this study did not have abnormal blood glucose levels. More importantly, GIP and derivatives can cross the blood brain barrier after peripheral administration (54). We synthesized long-acting GIPR agonist (D-Ala^2^-GIP) and antagonist (Pro^3^-GIP) (55). The analgesic and anxiolytic effects of D-Ala^2^-GIP were abolished by Pro^3^-GIP without affecting locomotor activity in male mice. In addition, treatment with D-Ala^2^-GIP or Pro^3^-GIP alone did not change the pain perception threshold or anxiety-like behaviors in control mice. These data suggest that the activation of GIPR only exerts analgesic and anxiolytic effects under pathological conditions rather than physiological conditions in male mice. However, the effects of GIPR in female mice still need to be confirmed.

The ACC, hippocampus, and amygdala are all involved in the regulation of chronic pain perception and the related emotional hang-ups (7, 56). After CFA injection, GIPR expression was increased in the ACC, but was not increased in the hippocampus or amygdala. GIPR activation had the analgesic and anxiolytic effects in the ACC, which seems to conflict with GIPR upregulation in the ACC of CFA-injected mice. The elevation of GIPR in the ACC may be attributed to the compensatory mechanism of self-analgesia. Our study first reported that GIPR in the ACC was involved in chronic inflammatory pain and pain-related anxiety modulation. This suggests that GIPR may be a potential target to treat chronic pain and accompanying mental disorders. Nevertheless, different chronic pain models may incorporate different grades of pain intensity, pain-related distress, and functional impairment (57). The role of GIPR in other brain regions and pain models needs to explore in the future.

Neuroinflammation is characterized by infiltration of immune cells, activation of glial cells, and production of inflammatory mediators in the peripheral and central nervous systems. Neuroinflammation also contributes to the induction and maintenance of chronic pain and anxiety (18, 19). In this study, our data showed that CFA-induced plantar edema did not subside on day 16, suggesting that there were continuous nerve impulses from local site of hindpaw to CNS. It has been previously reported that intraplanar injection of CFA could remarkably increase serum levels of IL-1β, IL-6, and TNF-α on day 4 (58). However, the serum levels of inflammatory factors did not increase significantly in this study, indicating that CFA-induced systemic inflammation may have been restored on day 16. Because of this possibility, we cannot completely exclude the role of peripheral cytokines in chronic pain and anxiety. Interestingly, D-Ala^2^-GIP, a GIPR agonist, significantly inhibited inflammation in the ACC of model mice. This was indicated by the regulation of GIPR on chemokine/receptor pairs CCL2/CCR2, CXCL1/CXCR2, and CXCL13/CXCR5, and pro-inflammatory cytokines IL-1β, IL-6, and TNF-α. Although the mRNA levels of CX3CL1/CX3CR1 and CCL4/CCR5 showed no difference in the ACC, they may play a role in the spinal cord and other brain regions at different times (29, 59). Microglia are a type of resident macrophage and principal immune-response cell in the CNS that are activated under pathological conditions. Microglia exhibit chemotactic, phagocytic, and secretory responses to various stimuli (60). Astrocytes can also produce pro-inflammatory cytokines and damage neurons (30). In our study, GIPR activation remarkably reversed the morphological changes of microglia, upregulations of Iba-1, and the levels of NF-κB p65 in the ACC. Neither CFA injection nor GIPR activation, alone or together, changed the morphology of astrocytes in the ACC or the expression of GFAP, which is an astrocyte activation marker. Obviously, microglia seem to be more susceptible than astrocytes, which was also implied in other reports (61). These results suggest that the analgesic and anxiolytic effects of GIPR are closely related to microglia and NF-κB p65 signaling.

The excitatory/inhibitory (E/I) neuronal network maintains a finely tuned balance that is critical for the physiological function of the CNS (48, 62). The imbalance of E/I signaling leads to a series of neurological disorders (63, 64). Enhanced excitatory transmission in the ACC contributes to both anxiety and nociception (65). Glutamate is the main excitatory neurotransmitter in the CNS. NMDA and AMPA receptors are two important subtypes of glutamate receptors, which are both related to chronic pain and anxiety (7, 66). In this study, activation of GIPR reversed the CFA-induced up-regulation of NMDA and AMPA receptors and enhanced both frequency and amplitude of mEPSCs and PPR reduction in the ACC. The frequency and amplitude of mEPSC represent presynaptic release and postsynaptic response, respectively. The decrease of PPR reflects an increase in release probability from excitatory presynapsis. Previous studies have indicated that CFA-induced chronic inflammatory pain could reduce GABAergic transmission in the ACC (67, 68), which is another reason for E/I imbalance. Whether GIPR activation produces analgesic and anxiolytic effects by enhancing GABAergic transmission needs to be further investigated. Together, our data suggest that the effects of GIPR are closely connected with inhibiting both the excitatory presynaptic release and postsynaptic responses. However, in line with other empirical results (69), CFA injection showed no effect on the spike numbers of Aps. This implies that the neuronal excitability is unchanged in the ACC after CFA and D-Ala^2^-GIP treatment.

**Figure 11:**
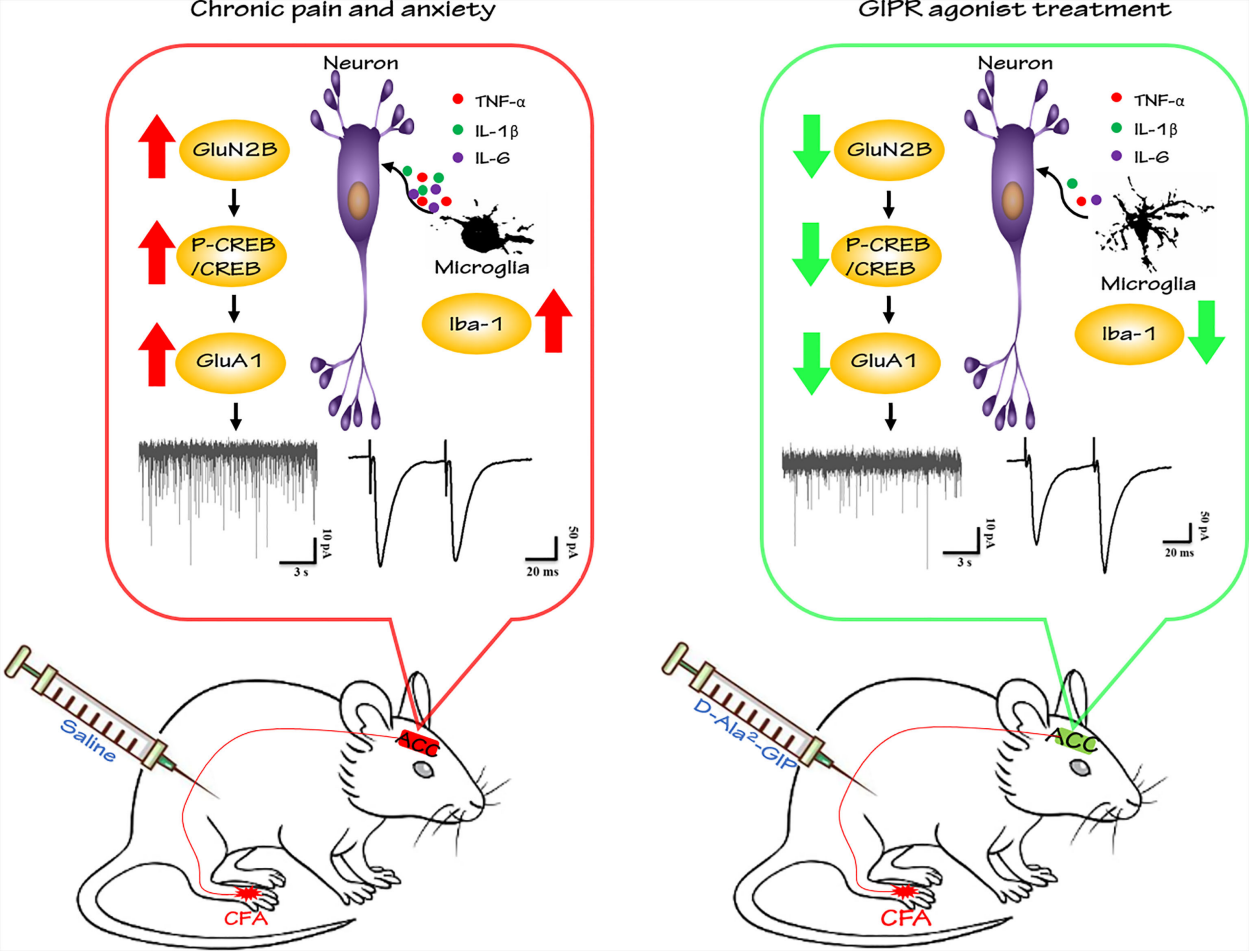
GIPR agonist exerts analgesic and anxiolytic-like effects in the ACC of mice with chronic inflammatory pain. In present study, hindpaw injection of CFA induced pain sensitization and anxiety-like behaviors in mice. D-Ala^2^-GIP (GIPR agonist) administration produced evident analgesic and anxiolytic effects by reversing microglia-induced neuroinflammation and suppressing the enhancement of excitatory neurotransmission in the ACC of model mice.

In summary, our study is the first to investigate the roles of GIPR in the ACC in the settings of chronic pain and pain-related anxiety. Our findings demonstrated the novel role of GIPR in the brain and support that GIPR is a good potential target to treat chronic inflammatory pain and pain-related anxiety. The effects of GIPR activation were partly due to attenuating neuroinflammation and inhibiting excitatory transmission in the ACC, as shown in **Figure 11**.

## Data Availability Statement

The original contributions presented in the study are included in the article/**Supplementary Material**. Further inquiries can be directed to the corresponding authors.

## Ethics Statement

The animal study was reviewed and approved by Animal Care and Use Committee of the Fourth Military Medical University.

## Author Contributions

S-bL, ZT, WJ, M-Gz, and X-sW conceived and performed experiments, wrote the manuscript, and secured funding. X-sW, Y-lJ, LL, BF, Q-yF, S-yG, and X-cZ performed experiments. KZ, FY, J-yQ, and L-kY performed statistical analysis. XM, LY, and X-bL provided expertise and feedback. All authors contributed to the article and approved the submitted version.

## Funding

This research was supported by grants from National Natural Science Foundation of China (No. 81801329, 82071474, 81771420, 31771119 and 82003734).

## Conflict of Interest

The authors declare that the research was conducted in the absence of any commercial or financial relationships that could be construed as a potential conflict of interest.

## Publisher’s Note

All claims expressed in this article are solely those of the authors and do not necessarily represent those of their affiliated organizations, or those of the publisher, the editors and the reviewers. Any product that may be evaluated in this article, or claim that may be made by its manufacturer, is not guaranteed or endorsed by the publisher.

## Abbreviations

GIP, Glucose-dependent insulinotropic polypeptide; CNS, the central nervous system; CFA, complete Freund’s adjuvant; GIPR, GIP receptor; ACC, anterior cingulate cortex; IBS, irritable bowel syndrome; IC, insular cortex; NMDA, N-methyl-D-aspartate; AMPA, α-amino-3-hydroxy-5-methyl-4-isoxazolepropionic acid; AD, Alzheimer’s disease; PD, Parkinson’s disease; AAV, adeno-associated virus; shRNA, short hairpin RNA; PWL, paw withdrawal latency; EPM, elevated plus maze; OF, open field; ELISA, Enzyme linked immuno-sorbent assay; ACSF, artificial cerebrospinal fluid; mEPSC, miniature excitatory postsynaptic current; PPR, paired-pulse ratio; Aps, action potentials; ANOVA, one-way analysis of variance; i.p., intraperitoneally; CREB, cAMP-response element binding protein; p-CREB, phosphorylated CREB; p-GluN2B-T1472, phosphorylated GluN2B at T1472; p-GluN2B-S1303, phosphorylated GluN2B at S1303; p-GluA1-S831, phosphorylated GluA1 at S831; p-GluA1-S845, phosphorylated GluA1 at S845.
