# Supplementary material for: Activation of GIPR Exerts Analgesic and Anxiolytic-Like Effects in the Anterior Cingulate Cortex of Mice
Source: Front Endocrinol (Lausanne). 2022 May 30;13:887238. doi: 10.3389/fendo.2022.887238 (PMC9196593; doi:10.3389/fendo.2022.887238)
Supplement: Supplementary file 1 [file DataSheet_1.pdf]

Supplementary Table. 1: The sequences of primers

Primer names	Sequences (Forward, 5' to 3')	Sequences (Reverse, 5' to 3')
CCL2	ACCTGCTGCTACTCATTCACC	CATTCCTTCTTGGGGTCAGCA
CCR2	CCTCAGTTCATCCACGGCAT	AGGGAGTAGAGTGGAGGCAG
CXCL1	GCCTCTAACCAGTTCCAGCA	TCTTGAGGTGAATCCCAGCC
CXCR2	GGGTCGTACTGCGTATCCTG	AGACAAGGACGACAGCGAAG
CXCL13	ACAAGAGGTTTGCGAGATGGA	CATGATGGCATTGCACCAGC
CXCR5	TCCATGCTGTTCACGCCTAC	GGCTCTAGTTTCCGCTTCGT
CX3CL1	GTGGCTTTGCTCATCCGCTA	GATGCTTCATGGCGTCTTGG
CX3CR1	TGCAGAAGTTCCCTTCCCATC	GGCCTCAGCAGAATCGTCATA
CCL4	CATGAAGCTCTGCGTGTCTG	GAGAAACAGCAGGAAGTGGGA
CCR5	TAGCCAGAGGAGGTGAGACA	GCAGGGTGCTGACATACCATA
TNF-α	TGTGCTCAGAGCTTTCAACAA	CTTGATGGTGGTGCATGAGA
IL-6	GACTGGGGATGTCTGTAGCTC	CAACTGGATGGAAGTCTCTTGC
IL-1β	TGCCACCTTTTGACAGTGATG	TGATGTGCTGCTGCGAGATT
β-actin	TGCTGTCCCTGTATGCCTCTG	TGATGTCACGCACGATTTCC

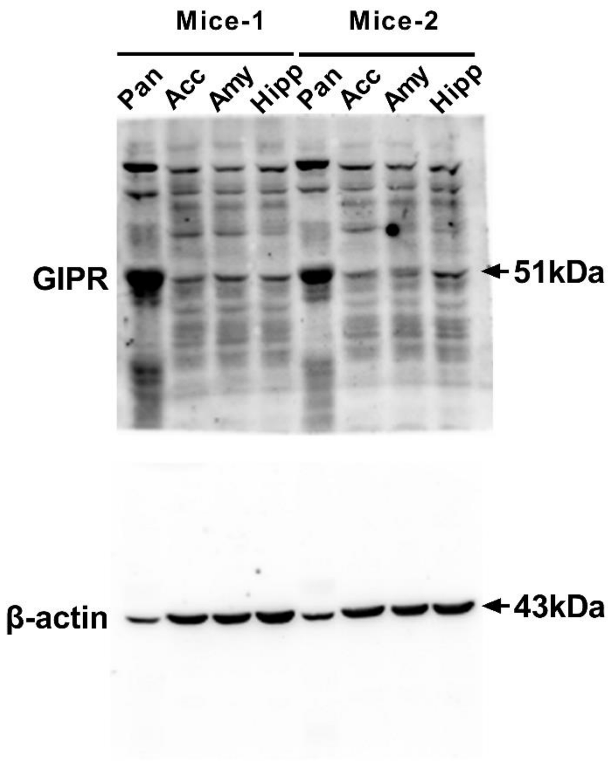

S-Fig. 1 Location of GIPR on PVDF membrane and expression of GIPR in the limbic system

Pancreas (Pan), ACC, amygdala (Amy) and hippocampus (Hipp) tissues were obtained from two mice without any treatment. Western-blot analysis was employed and the location of GIPR was identified at 51 kDa on the entire PVDF membrane. GIPR is expressed in pancreas, ACC, amygdala and hippocampus. Pan was used as positive control.

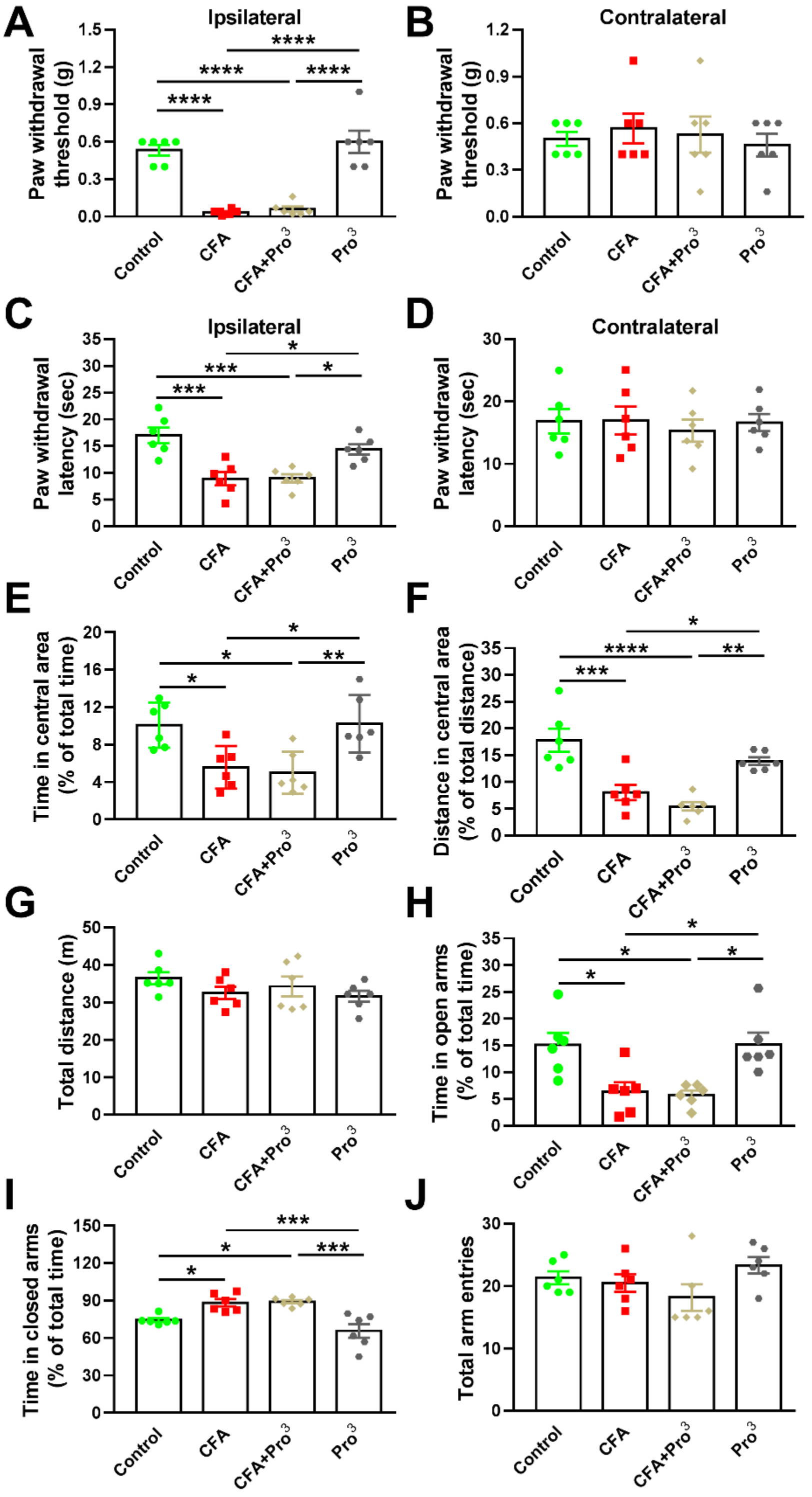

S-Fig. 2 No effects of Pro^3-GIP on nociceptive and anxiety-like behaviors

Administration of Pro^3-GIP (25 nM/kg, i.p., once daily for 10 days) alone did not affect paw withdrawal threshold **(A, B)** and latency **(C, D)**. Pro^3-GIP alone also had no effects on the anxiety-like behaviors in CFA-injected mice and control mice in OF test **(E-G)** and EPM test **(H-J)**. n = 6 in each group. Totally, 24 mice were used in this section. Pro^3 means Pro^3-GIP. $^{*}p < 0.05$, $^{***}p < 0.001$, $^{****}p < 0.0001$.

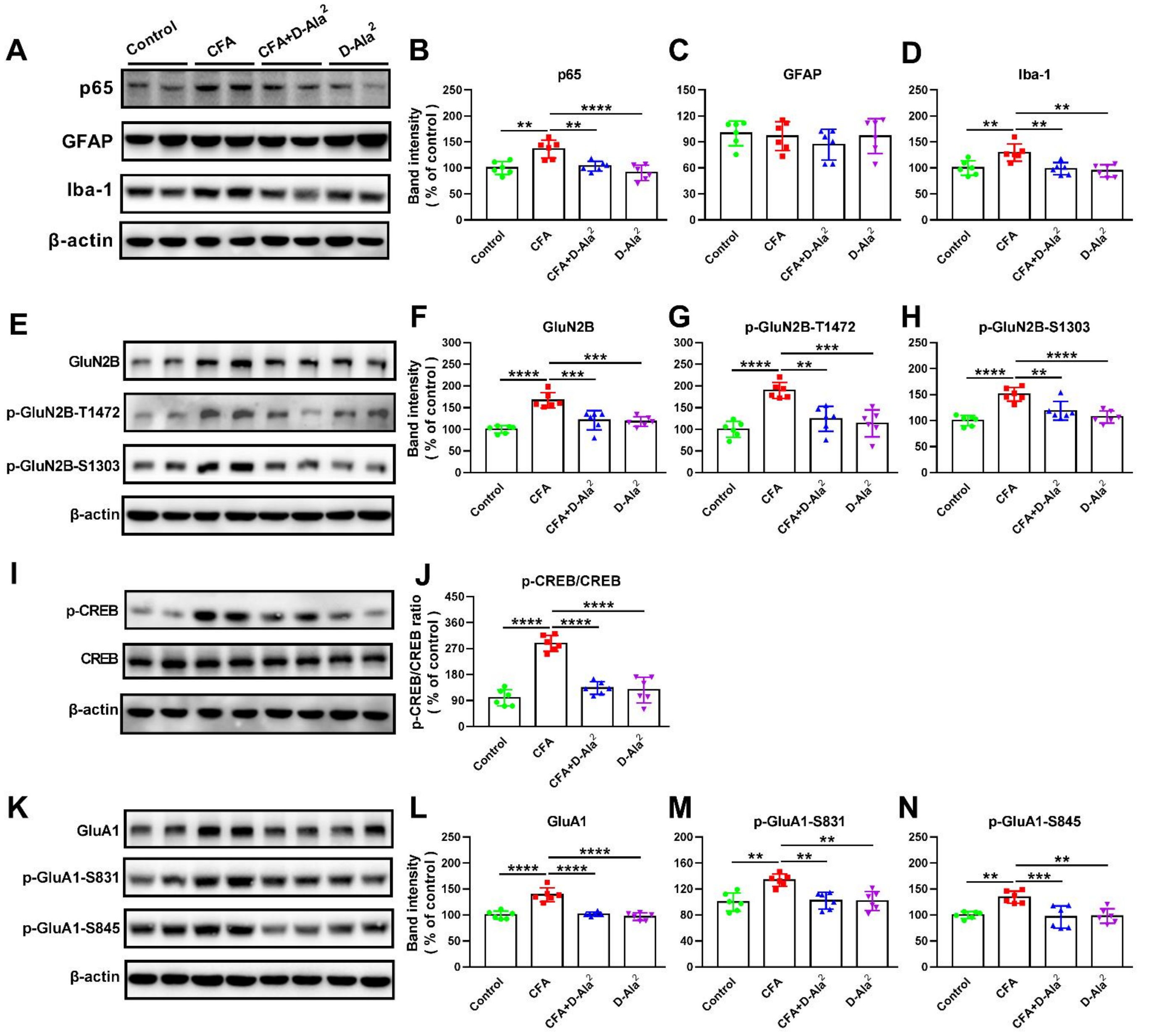

S-Fig. 3 Effects of GIPR activation on the levels of pain-related proteins in the ACC

(A) Representative Western blot analysis of NF-κB p65, GFAP and Iba-1. **(B-D)** CFA injection significantly increased NF-κB p65 **(B)** and Iba-1 **(D)** levels in the ACC, but not GFAP level **(C)**. D-Ala^2-GIP notably reversed the increased expressions of NF-κB p65 and Iba-1. **(E)** Representative Western blot analysis of GluN2B, p-GluN2B-T1472 and p-GluN2B-S1303. **(F-H)** Systemic administration of D-Ala^2-GIP reduced the increased expressions of GluN2B, p-GluN2B-T1472 and p-GluN2B-S1303 in the ACC of CFA-injected mice. **(I)** Representative Western blot analysis of p-CREB and CREB. **(J)** D-Ala^2-GIP inhibited the increase of p-CREB/CREB ratio induced by CFA injection. **(K)** Representative Western blot analysis of GluA1, p-GluA1-S831 and p-GluA1-S845. **(L-N)** D-Ala^2-GIP treatment reversed the up-regulations of GluA1, p-GluA1-S831 and p-GluA1-S845 in the ACC of CFA-injected mice. n = 6 in each group. Totally, 24 mice were used in this section. D-Ala^2 means D-Ala^2-GIP. $^{**}p < 0.01$, $^{***}p < 0.001$, $^{****}p < 0.0001$.
